# The role of conformational flexibility in β_2_-microglobulin amyloid fibril formation at neutral pH

**DOI:** 10.1002/rcm.6282

**Published:** 2012-07-02

**Authors:** John P Hodkinson, Sheena E Radford, Alison E Ashcroft

**Affiliations:** 1 Astbury Centre for Structural Molecular Biology, Institute of Molecular and Cellular Biology, Faculty of Biological Sciences, University of LeedsLeeds, LS2 9JT UK

## Abstract

**RATIONALE** Amyloid formation is implicated in a number of human diseases. β_2_-microglobulin (β_2_m) is the precursor protein in dialysis-related amyloidosis and it has been shown that partial, or more complete, unfolding is key to amyloid fibril formation in this pathology. Here the relationship between conformational flexibility and β_2_m amyloid formation at physiological pH has been investigated.

**METHODS** HDX-ESI-MS was used to study the conformational dynamics of β_2_m. Protein engineering, or the addition of Cu^2+^ ions, sodium dodecyl sulphate, trifluoroethanol, heparin, or protein stabilisers, was employed to perturb the conformational dynamics of β_2_m. The fibril-forming propensities of the protein variants and the wild-type protein in the presence of additives, which resulted in >5-fold increase in the EX1 rate of HDX, were investigated further.

**RESULTS** ESI-MS revealed that HDX occurs via a mixed EX1/EX2 mechanism under all conditions. Urea denaturation and tryptophan fluorescence indicated that EX1 exchange occurred from a globally unfolded state in wild-type β_2_m. Although >30-fold increase in the HDX exchange rate was observed both for the protein variants and for the wild-type protein in the presence of specific additives, large increases in exchange rate did not necessarily result in extensive *de novo* fibril formation.

**CONCLUSIONS** The conformational dynamics measured by the EX1 rate of HDX do not predict the ability of β_2_m to form amyloid fibrils *de novo* at neutral pH. This suggests that the formation of amyloid fibrils from β_2_m at neutral pH is dependent on the generation of one or more specific aggregation-competent species which facilitate self-assembly. Copyright © 2012 John Wiley & Sons, Ltd.

The ability to form amyloid fibrils, a generic property of possibly all polypeptide chains, is driven by main-chain/main-chain interactions, which are modulated by the sequence of amino acid side-chains.^1^ In natively structured proteins, aggregation-prone sequences are usually sequestered from solvent by the tertiary structure of the protein, with the result that self-assembly can only take place once unfolding from the native state occurs and exposes the aggregation-prone region.^2^ This idea is known as the 'conformational change hypothesis' and is supported by the fact that amyloid fibril precursors can exhibit all types of secondary structure organisation, whereas amyloid fibrils exhibit a characteristic, common cross-beta structure.^3^ Examples supporting the conformational change hypothesis in which a native protein undergoes significant re-organisation to form the amyloid fold include transthyretin,^4^ lysozyme,^5^ prion^6^ and light chain amyloidoses.^7^ Consistent with this, *in vitro* studies have shown that denaturation or destabilisation of the native state of globular proteins can increase amyloidogenicity.^1^,^2^,^4^–^9^

β_2_-microglobulin (β_2_m) is a 99 amino acid protein that exhibits a typical immunoglobulin fold comprising seven β-strands which form an anti-parallel β-sandwich structure stabilised by an inter-strand disulphide bridge between residues 25 and 80 (Fig. 1).^10^,^11^ The β_2_m sequence contains five proline residues, of which four are in the *trans* conformation, and one, proline 32, is in the *cis* conformation in the natively folded protein.^10^ β_2_m has an important physiological role as the light chain of the major histocompatibility complex class I (MHC-1) and its sequence has evolved to optimise its role as a chaperone during MHC-1 assembly.^12^ This evolutionary pressure for function has resulted in a sequence with amyloid propensity when dissociated from the heavy chain of this complex.^9^,^13^,^14^ Normally, β_2_m is released from the MHC-1 whereupon it is degraded by the kidney. However, in renal insufficiency, β_2_m is not degraded and its concentration in serum increases 25- to 60-fold, with the ultimate result that the protein aggregates into insoluble amyloid fibrils in the joints, resulting in a pathological condition termed dialysis-related amyloidosis (DRA).^15^

**Figure 1:**
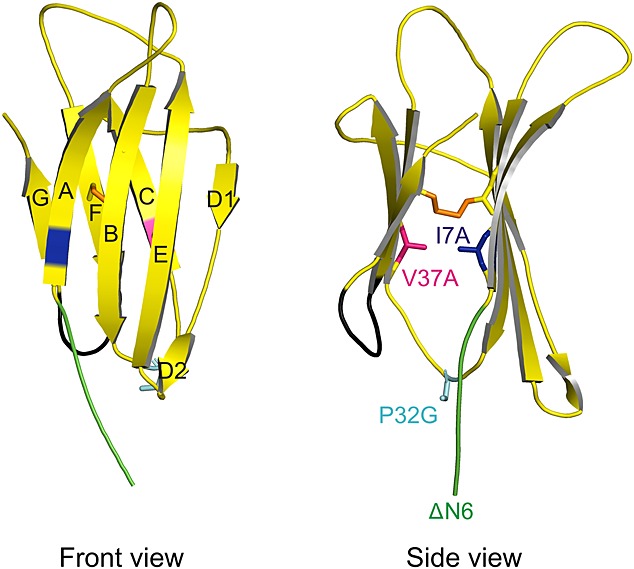
The solution structure (PDB accession code 1JNJ)^11^ of wild-type human β_2_m. The residues/regions mutated in the four variants in these studies are highlighted: ΔN6 (green), I7A (dark blue), P32G (cyan) and V37A (pink). The position of the FG loop that differs markedly in sequence between human and murine β_2_m^29^ is shown in black. The β-strands are annotated A–G and the disulphide bond is shown as an orange stick linking strands B and F.

Akin to other proteins which form amyloid fibrils commencing from a stably folded native structure, conformational change is also necessary for amyloid formation from β_2_m.^16^,^17^ It has been shown that, at physiological temperature and pH, wild-type human β_2_m does not form amyloid fibrils spontaneously *in vitro*, even when incubated for long periods of time at high protein concentration.^18^,^19^ This finding led to the investigation of alternative conditions under which β_2_m could be induced to form amyloid fibrils *in vitro* and the discovery that at acidic pH, β_2_m rapidly and extensively assembles into fibrils that possess all the characteristic structural, tinctorial and ligand binding properties of amyloid.^20^–^22^ Given that 60% of the sequence of β_2_m is predicted to be highly amyloidogenic by various algorithms and by studies of isolated peptides,^9^ it is not surprising that unfolding the protein with acidic pH results in rapid fibril formation.^21^,^22^ These observations support the view that conditions or mutations that increase the conformational flexibility of the protein will also enhance its amyloidogenic potential.^13^,^23^–^26^

The conformational flexibility of wild-type human β_2_m under native conditions has been investigated by hydrogen deuterium exchange (HDX) monitored by electrospray ionisation mass spectrometry (ESI-MS), capillary electrophoresis and nuclear magnetic resonance (NMR).^13^,^23^,^24^,^26^,^27^ HDX-ESI-MS is particularly well suited to uncovering unfolded species within a native protein ensemble because exchange arising from uncorrelated conformational fluctuations (EX2) can be differentiated from exchange arising from transient, large-scale unfolding events (EX1) under appropriate conditions. From these studies, an unprotected conformer detected through EX1 exchange at pH 7 has been implicated in β_2_m amyloid fibril formation under native conditions.^13^,^23^,^24^,^26^ However, the nature of this unprotected species remained unclear. Here we investigate the role of the conformational dynamics of β_2_m in the formation of amyloid fibrils under native-like conditions (pH 7, 37 °C). We show that hydrogen exchange from the native protein (N) under these conditions occurs by a mixed EX1/EX2 regime in which the native protein is in equilibrium with partially protected conformers, as well as the globally unfolded state (U). In addition, we use protein engineering, together with the addition of Cu^2+^ ions, trifluoroethanol (TFE), sodium dodecyl sulphate (SDS), low molecular weight (LMW) heparin or the protein stabilisers, trimethyl amine oxide (TMAO), sarcosine and trehalose, to modulate the conformational dynamics of the wild-type protein visualised by the EX1 rate of exchange and then to determine whether amyloid fibril formation is observed under the conditions selected. The results show that although the EX1 rate of exchange can be varied by up to 35-fold by means of the additives and mutations described, a substantial increase in the EX1 rate alone does not predict whether amyloid fibrils will be formed *de novo* within a set timescale.

## EXPERIMENTAL

### Mutagenesis and protein purification

Wild-type human β_2_m and its variants were expressed and purified as described previously.^28^ Murine β_2_m was expressed and purified as described previously.^29^

### D→H exchange of wild-type β_2_m and variants in the presence of Cu^2+^/TFE/SDS or stabilisers monitored by ESI-MS

Fully deuterated protein^13^ (lyophilised β_2_m dissolved in 99.9% D_2_O buffered to pH 8.0 using 5 mM TrisHCl and made up to a final protein concentration of 600 μM) was diluted 25-fold into protonated 10 mM ammonium formate/10 mM ammonium acetate buffer containing the appropriate additive at pH 7.0 to initiate HDX. The solution was mixed using a vortex mixer and returned to a water bath at the appropriate temperature. An aliquot (200 μL) of the reaction mixture was removed at selected time-points, loaded onto a 5 mL HiTrap desalting column (GE Healthcare Life Sciences, Amersham, UK), and eluted rapidly with the same buffer (without additives). The eluate was transferred to the NanoMate (Advion Biosciences Inc., Ithaca, NY, USA) autosampler and nanoESI interface of the mass spectrometer. The sample was ionised and desolvated by nanoESI (nitrogen nebulising gas pressure 0.4 psi, capillary voltage 1.8 kV), and analysed by use of an LCT Premier time-of-flight (TOF) mass spectrometer (Waters UK Ltd., Manchester, UK) with the following instrument settings: cone voltage: 70 V; ion guide: 70 V; aperture: 10 V; ion energy: 100 eV.

### Analysis of the ESI-MS data

**Figure 2:**
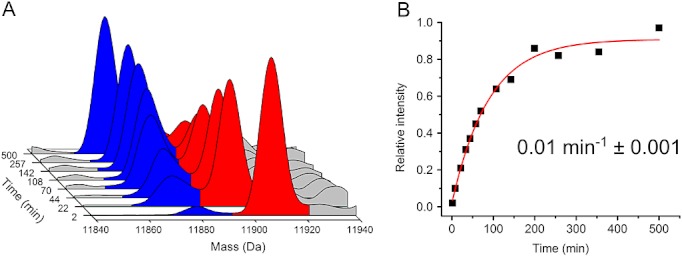
HDX-ESI-MS of wild-type β_2_m. (A) D→H exchange of wild-type β_2_m (pH 7.0, 37 °C). Protein molecules which have undergone exchange *via* an EX2 mechanism only are shown on the right in red, while those which have undergone exchange *via* both an EX2 and EX1 mechanism are shown on the left in blue. (B) Plot showing the intensity of the EX1 peak relative to the total protein signal over time. The data are fitted to a single exponential (red line).

ESI-MS data were processed with the MassLynx 4.1 software supplied with the mass spectrometer (Waters UK Ltd.). The data were smoothed twice with the Savitsky-Golay algorithm and centred by peak area. Each spectrum was fitted to two sets of Gaussian curves centred on the EX1 and EX2 base peaks with Igor Pro v5.4 software (Wavemetrics Inc., Portland, OR, USA). The base peaks were linked to three additional peaks: base peak minus 28 *m/z* units, base peak plus 22 *m/z* units (Na^+^ adduct) and base peak plus 38 *m/z* units (K^+^ adduct). The peak positions of the adducts were determined in each spectrum by linking them to the base peak (±0.2 *m/z* units). The adduct peak width was fixed relative to the base peak (±0.1 *m/z* units) and the adduct peak height was fixed according to the ratio relative to the base peak observed in the initial and final spectra (±10%). The area and mass of each peak were determined at each time-point during the HDX reaction by use of this method. For the data shown in Figs. 2 and 4, the *m/z* spectra were transposed onto a mass (Da) scale using the Maximum Entropy software supplied with the mass spectrometer.

### Unseeded fibril growth at pH 7.0 in the presence of additives

Each protein solution (84 μM) containing 0.02% (w/v) sodium azide, 10 μM thioflavin T (ThT), the required concentration of additive and 10 mM ammonium formate/10 mM ammonium acetate buffer was brought to pH 7.0 with hydrochloric acid and then passed through an Anotop 0.2 µm syringe filter (Whatman UK Ltd., Maidstone, UK). A small volume of the solution (50 μL/well) was pipetted into a 384-well plate with the required number of replicates. The plates were sealed with clear plastic film. The fluorescence of ThT was excited (444 nm), and the emitted fluorescence measured at 480 nm at hourly time-intervals at 37 °C with a Fluostar Optima plate reader (BMG Labtech GmbH, Offenburg, Germany) with continuous shaking at 200 rpm.

A β_2_m concentration of 84 μM was found to produce fibrils in an experimentally tractable time-frame under the conditions employed. As previous work^30^ has indicated that there are no differences in the fibrils generated over a concentration range of 8–244 μM under the conditions employed, the difference between this protein concentration and that most suitable for the ESI-MS analyses (24 μM) was not of any concern.

### Electron microscopy (EM)

An aliquot of sample at the end of each fibril growth assay (14 days) was pipetted onto a sheet of parafilm. A freshly ionised formvar/carbon-coated EM grid was placed with the coated side onto the droplet and allowed to stand for 30 s. Remaining sample solution was removed with filter paper and 10 μL of 4% (w/v) uranyl acetate solution was pipetted onto the parafilm. The grid was again placed coated side down onto the droplet for 30 s. Excess uranyl acetate solution was removed with filter paper and the grid was air-dried. Electron micrographs of the samples were taken with a JEOL 1200 electron microscope (JEOL Ltd., Tokyo, Japan) at a magnification that depended on the sample being analysed.

### Determination of the unfolding rate of human wild-type β_2_m

A volume of 100 μL of 84 μM protein in 10 mM ammonium formate/10 mM ammonium acetate buffered to pH 7 was mixed with the appropriate volume of 9 M urea dissolved in the same buffer mixture to yield the required final urea concentration at 37 °C. This mixture was immediately transferred into a pre-warmed cuvette. The cuvette contained a magnetic stirring bar which was used at 200 rpm during the analysis of the samples. The fluorescence of the protein was measured at 350 nm (using excitation at 280 nm) for 12 min or until the signal reached equilibrium in a QuantaMaster UV/VIS fluorescence spectrometer (Photon Technology International Inc., Birmingham, NJ, USA).

## RESULTS

### β_2_m visits a globally unfolded conformation under physiological conditions

It has been shown previously that HDX of native wild-type human β_2_m at pH 7, 37 °C, occurs by a mixed EX1/EX2 mechanism.^13^,^24^,^26^ To investigate the mechanism of HDX of β_2_m further, and specifically to determine whether exchange *via* the EX1 regime reflects global or sub-global unfolding events, the fully deuterated protein was diluted 25-fold into protonated buffer at pH 7, 37 °C, and exchange was monitored by ESI-MS (see Experimental section). Consistent with previous results,^13^,^24^ the spectra show that deuteron exchange (D→H) from the native, protected state occurs in part by local/uncorrelated motions (which result in a gradual decrease in mass of the fully protected species indicative of an EX2 mechanism of exchange; Fig. 2(A)). In addition, HDX occurs concurrently by correlated conformational fluctuations to a species that initially protects only 20 deuterons from exchange (characteristic of an EX1 mechanism of exchange; Fig. 2(A)). These residual 20 deuterons subsequently exchange *via* an apparent EX2 mechanism until exchange is complete (Fig. 2(A)). This behaviour has been observed for other proteins^31^,^32^ and, in a two-state protein, may result from residual protection in the unfolded state, or competition between k_f_ (the refolding rate constant) and k_int_ (the rate constant of exchange from the unprotected state).

To determine the rate of HDX occurring by the EX1 mechanism, the spectra were integrated and software deconvolution was employed to determine the mass and population of the different species formed *versus* time, taking account of any ion adducts to each species (see Experimental section). The results revealed that exchange to the unprotected state occurs with a rate constant of 0.01 ± 0.001 min^–1^ (Fig. 2(B)), consistent with a value of 0.009 min^–1^ reported previously.^24^ To illuminate whether the apparent EX2 exchange of the 20 protected residues results from residual structure in the unfolded state or competition between k_f_ and k_int_, the pH dependence of the apparent EX2 exchange was investigated. An increase in the pH by one unit should increase k_int_ 10-fold. If the phenomenon described results from rate competition, as k_int_ is increased the extent of proton exchange by apparent EX2 kinetics should decrease. The data showed that as the pH is reduced from pH 7 to pH 5 the mass of the post EX1 exchange ion (Fig. 2(A)) increased by 7 Da units. Similarly, as the pH is increased from pH 7 to pH 9 the magnitude of the apparent EX2 shift of this peak reduced to zero. The results suggest, therefore, that the protein is visiting a globally unprotected conformation across the pH range 5–9, but it is only at pH 9 that the k_int_ is sufficiently rapid, relative to the refolding rate constant, to ensure complete exchange of all exposed residues upon visiting this globally unprotected state.

**Figure 3:**
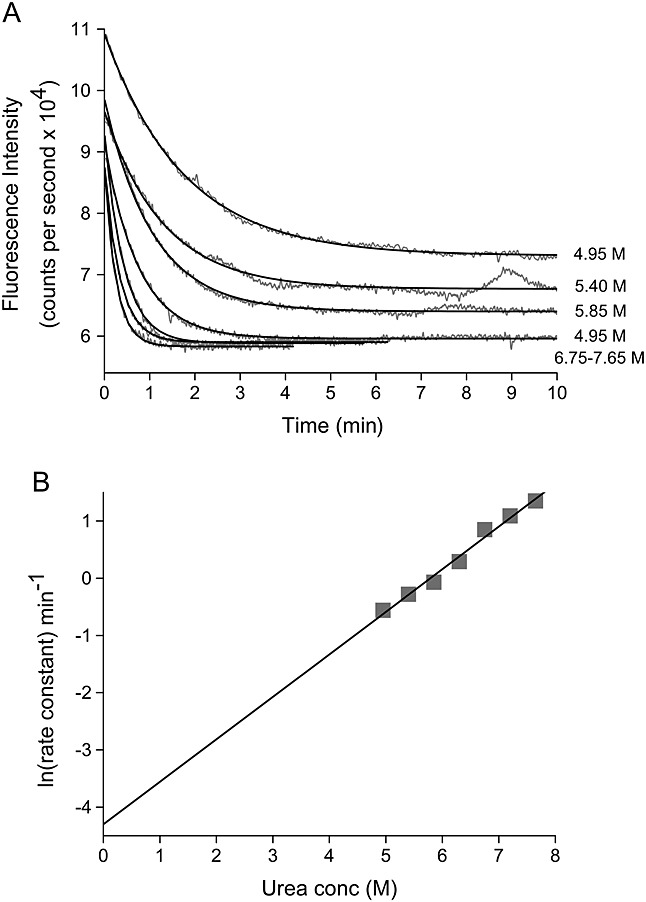
Determination of wild-type β_2_m unfolding rate. (A) Plot showing the unfolding of wild-type human β_2_m measured using tryptophan fluorescence (pH 7.0, 37 °C) at increasing urea concentration (4.95-7.65 M urea in 0.45 M increments). The data are shown in grey and a single exponential fit to the data is shown as a black line. (B) Plot of the natural logarithm of the unfolding rate constant of wild-type human β_2_m (pH 7.0, 37 °C) vs. the urea concentration (M). The data are shown as grey squares and the linear fit to the data in black. The error on each point (2 standard deviations of the mean) is smaller than the data marker.

To determine whether the unprotected state of β_2_m visualised by EX1 exchange is globally unfolded, or represents a more structured yet unprotected state, the unfolding rate constant (k_u_) of β_2_m was measured by dilution of the native protein into high concentrations of urea and the rate of unfolding monitored by the fluorescence of tryptophan residues (Fig. 3(A)). The data revealed that the protein unfolds by a single exponential function and that the logarithm of the rate constant of unfolding depends linearly on the concentration of denaturant (Fig. 3(B)). Extrapolation of these data yields an unfolding rate constant in the absence of denaturant of 0.01 ± 0.001 min^–1^, a value identical to the rate of EX1 exchange measured by HDX-MS under these conditions (Figs. 2(B) and (B)). The results indicate that exchange by EX1 kinetics results from global unfolding and that native β_2_m visits a globally unfolded, highly unprotected conformational state transiently under conditions in which the protein appears compactly folded.^11^,^17^,^33^ The following analyses, therefore, focused on investigating the EX1 exchange rate to explore the importance of kinetic stability and the probability of global unfolding for the self-aggregation of β_2_m at neutral pH. These data also pose the intriguing question of why evolution has allowed β_2_m to retain a high level of kinetic instability and to sample the unfolded state regularly, which would ordinarily pose a risk of aggregation.^34^

### Varying the frequency of visiting the unfolded state with solvent additives or protein engineering

To explore the possibility that global unfolding of native β_2_m at pH 7.0 determines the ability of the protein to form amyloid fibrils at neutral pH, conditions were explored that enhanced or reduced the probability of global unfolding, as determined by the rate of EX1 HDX, and the effect of these alterations in protein dynamics on fibril formation was monitored in parallel. These conditions involved the use of β_2_m variants or incubating the wild-type protein in buffer at pH 7.0 in the presence of additives that alter the stability of the native state. In each case, the EX1 HDX rate was determined at pH 7.0 by ESI-MS. The variants selected for analysis were chosen from an array previously shown to display a wide variation in thermodynamic stability and amyloid potential: namely I7A, V37A, P32G, and ΔN6 (Fig. 1).^16^,^19^,^28^,^35^–^37^ Specifically, the mutation I7A truncates a buried aliphatic side chain and destabilises β_2_m by 6 kJ mol^–1^ (ΔG^0^_UN_ = −10.3 kJ mol^–1^ compared with ΔG^0^_UN_ = −16.5 kJ mol^–1^ for wild-type β_2_m at pH 7.0, 37 °C). This sequence alteration results in a protein able to form fibrils at pH 7.0 *in vitro*, albeit with low yield.^28^,^37^ V37A destabilises β_2_m substantially (ΔG^0^_UN_ = −4.7 kJ mol^–1^ at pH 7.0, 37 °C), yet does not enable fibril formation at this pH.^19^,^28^ P32G is mildly destabilised (ΔG^0^_UN_ = −11.4 kJ mol^–1^ at pH 7.0, 37 °C) and enhances the ability of β_2_m to elongate fibril seeds, but this protein cannot nucleate fibril assembly at neutral pH.^16^ The variant ΔN6 (a protein which lacks the N-terminal six amino acids) was included in the analysis as this protein is also mildly destabilised (ΔG^0^_UN_ = −12.0 kJ mol^–1^ at pH 7.0, 37 °C) and, uniquely amongst the set of protein sequences studied, is able to nucleate fibril formation efficiently at neutral pH.^33^,^35^,^36^,^38^ Finally, murine β_2_m was analysed because this protein, despite sharing 70% sequence identity with human β_2_m,^29^,^37^ lacks the ability to form amyloid fibrils under acidic or neutral pH conditions.

In parallel with the mutagenesis studies, wild-type β_2_m was also incubated in the presence of a selection of additives, which have been reported to endow a range of effects on aggregation propensity. First, the divalent metal ions Cu^2+^, Zn^2+^ and Ni^2+^ were added. Cu^2+^ has been reported to facilitate amyloid fibril formation *de novo* from wild-type β_2_m, while the latter two ions have no effect, despite their similar valence and size.^8^,^38^,^39^ The co-solvent TFE (5–20% (v/v)) was also employed because this solvent has been shown to enhance elongation of GAG-stabilised β_2_m seeds^40^,^41^ and to promote the population of non-native conformers when added at a concentration of 5–20% (v/v) at neutral pH.^40^–^43^ SDS (0.5–2 mM) was included in this analysis as this anionic surfactant has been shown to promote partial unfolding of β_2_m and to promote the elongation of fibril seeds at concentrations below its critical micellar concentration.^44^ LMW heparin was included as a control additive, as this glycosaminoglycan has been shown to stabilise fibrils but to have little effect on the structure or stability of the β_2_m monomer.^19^ Additives that would be expected to stabilise native β_2_m and reduce the probability of unfolding were also included. Accordingly, trehalose, sarcosine and trimethylamine *N*-oxide (TMAO) were also used in these studies. These protein stabilisers were chosen for their known effect on the thermodynamic or kinetic stability of folded proteins and the consequent reduction in their aggregation propensity.^45^,^46^ The choice of additives, solvents and sequence variants was designed to enable a systematic analysis of the HDX properties of β_2_m and, thereby, to determine if the dynamics of folded β_2_m correlate with the amyloidogenicity of the protein monomers.

**Figure 4:**
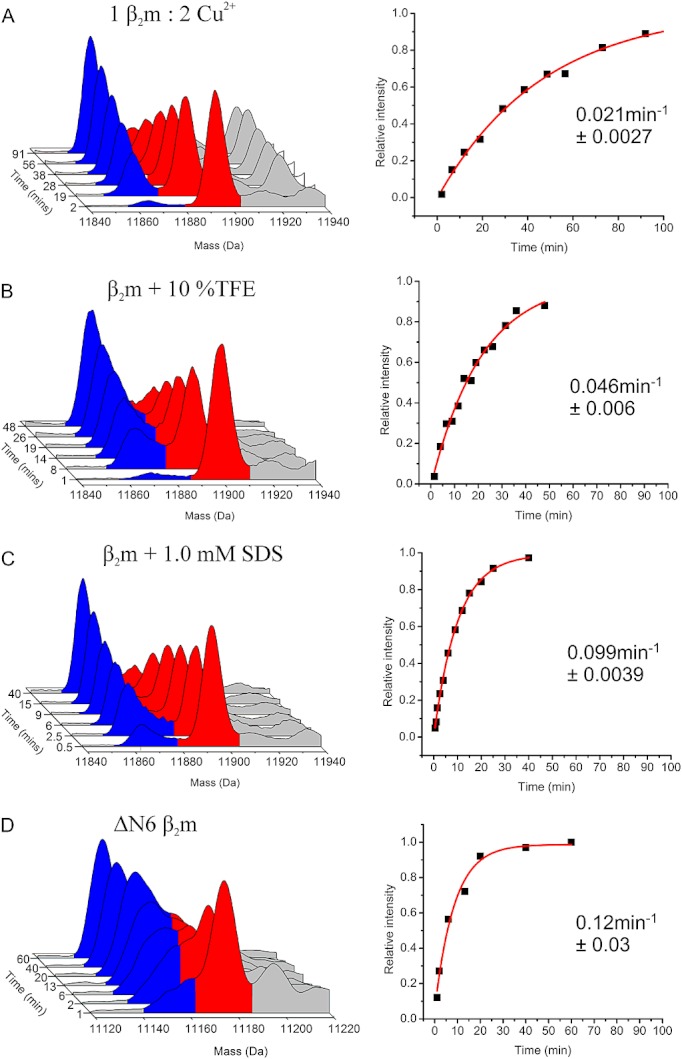
HDX-ESI-MS of wild-type β_2_m in presence of Cu^2+^, TFE or SDS and also of ΔN6 β_2_m. D→H exchange of wild-type human β_2_m (pH 7.0, 37 °C) in the presence of (A) a 2-fold molar excess of Cu^2+^ ions; (B) 10 % (v/v) TFE; and (C) 1 mM SDS. (D) D→H exchange of ΔN6 β_2_m (pH 7.0, 22 °C). In each case the left-hand image shows the mass profile of the ESI-MS spectrum. Protein molecules which have undergone exchange *via* an EX2 mechanism only are shown on the right in red, while peaks that arise from molecules which have undergone exchange *via* both EX2 and EX1 mechanisms are shown on the left in blue. Peaks in (A) and (D) at higher mass (grey) result from potassium and sodium adducts. The right-hand column shows plots of EX1 peak intensity relative to the total protein signal over time. The data are fitted to a single exponential (red line) in each case.

**Figure 5:**
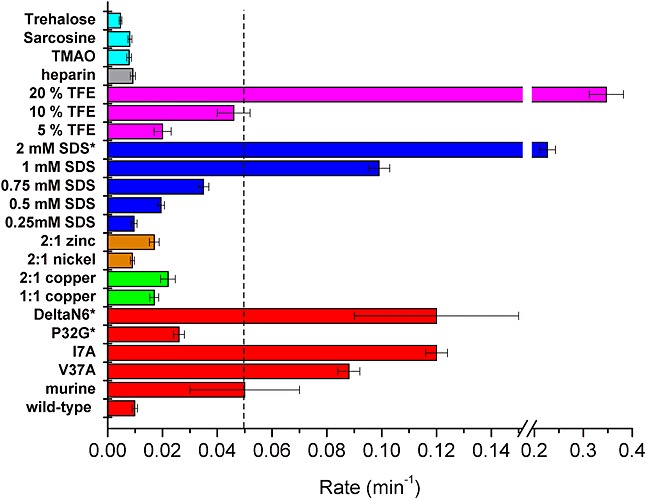
The EX1 rate of HDX of wild-type human β_2_m, its variants and murine β_2_m in the presence of a range of different solution conditions. All reactions were measured at pH 7.0, 37 °C, except for those marked with * which were carried out at 22 °C. The error bars depict two standard deviations of the mean. The dashed line denotes the threshold of a rate of HDX ≥ 5–fold that of wild-type human β_2_m.

Examples of the HDX kinetics of the different variants of β_2_m and the wild-type protein in the presence of different additives are shown in Fig. 4. Under all the conditions explored, the proteins exchanged with mixed EX1/EX2 kinetics as shown for the wild-type protein in buffer alone (Fig. 2). However, despite undergoing exchange by a similar mechanism, the rate of EX1 exchange varied widely throughout the different sequences and conditions used (Fig. 5 and Table 1). For example, the addition of SDS (at concentrations >0.5 mM) or TFE (at concentrations >5% (v/v)) resulted in a large (up to 35-fold) increase in the rate of HDX occurring by an EX1 mechanism. The addition of 2 mM SDS resulted in a >20-fold increase in the rate of EX1 HDX kinetics, while the inclusion of 20% (v/v) TFE increased the EX1 rate of HDX by 35-fold. A large increase in EX1 HDX kinetics was also apparent for the variants ΔN6 and P32G β_2_m for which the rates of HDX were too rapid to measure at 37 °C. Thus, for these variants, the temperature was reduced to 22 °C in order to obtain accurate measurement of their HDX rates (Table 1 and Fig. 5). All the variants tested exhibited increased conformational flexibility relative to wild-type β_2_m as judged by the rate of HDX, with murine β_2_m showing a rate of EX1 HDX 5-fold greater that than of the human protein, while V37A and I7A showed EX1 HDX rates which were increased by 9- and 12-fold, respectively, relative to the wild-type human protein. Interestingly, the most kinetically unstable β_2_m variants, (ΔN6 and P32G) were not the most thermodynamically unstable proteins (V37A and I7A β_2_m), indicating a lack of correlation of the kinetic and thermodynamic stabilities of this protein. A modest increase (2-fold) in the EX1 rate of HDX was observed in the presence of a 2-fold molar excess of Cu^2+^, while little, or no, increase (<2-fold) occurred in the reactions containing a 2-fold molar excess of Ni^2+^ or Zn^2+^. Finally, no changes in the HDX properties of wild-type β_2_m were discernible in reactions incorporating LMW heparin, while up to 2-fold reduction in the rate of EX1 HDX was evident upon addition of protein stabilisers, with the greatest effect observed with trehalose. Armed with this wide range of distinct effects on the dynamic properties of β_2_m, we aimed to uncover whether kinetic instability is a generic basis for the amyloid propensity of this protein.

**Table 1:** Rate of EX1 HDX of wild-type human β_2_m, its variants, and murine β_2_m at pH 7.0, 37 °C, in the presence of a range of additives

Additive/β_2_m variant	EX1 HDX rate (min^–1^)
Trehalose (500 mM)	0.0047 ± 0.00051
Sarcosine (250 mM)	0.0081 ± 0.00072
TMAO (250 mM)	0.0078 ± 0.00086
LMW heparin (168 μM)	0.0092 ± 0.00096
20 % (*v/v*) TFE	0.347 ± 0.035
10 % (*v/v*) TFE	0.046 ± 0.006
5 % (*v/v*) TFE	0.02 ± 0.0031
2.0 mM SDS*	0.227 ± 0.016
1.0 mM SDS	0.099 ± 0.0039
0.75 mM SDS	0.035 ± 0.0019
0.5 mM SDS	0.0195 ± 0.0012
0.25 mM SDS	0.0096 ± 0.0011
β_2_m 1:2 Zn^2+^	0.017 ± 0.0018
β_2_m 1:2 Ni^2+^	0.009 ± 0.00075
β_2_m 1:2 Cu^2+^	0.021 ± 0.0027
β_2_m 1:1 Cu^2+^	0.017 ± 0.0016
ΔN6 β_2_m*	0.12 ± 0.03
P32G β_2_m*	0.026 ± 0.002
I7A β_2_m	0.12 ± 0.03
V37A β_2_m	0.088 ± 0.03
Murine β_2_m	0.05 ± 0.02
Human β_2_m (wild-type)	0.01 ± 0.001

* These reactions were carried out at 22 °C.

### The probability of global unfolding does not correlate with the probability of fibril formation

**Figure 6:**
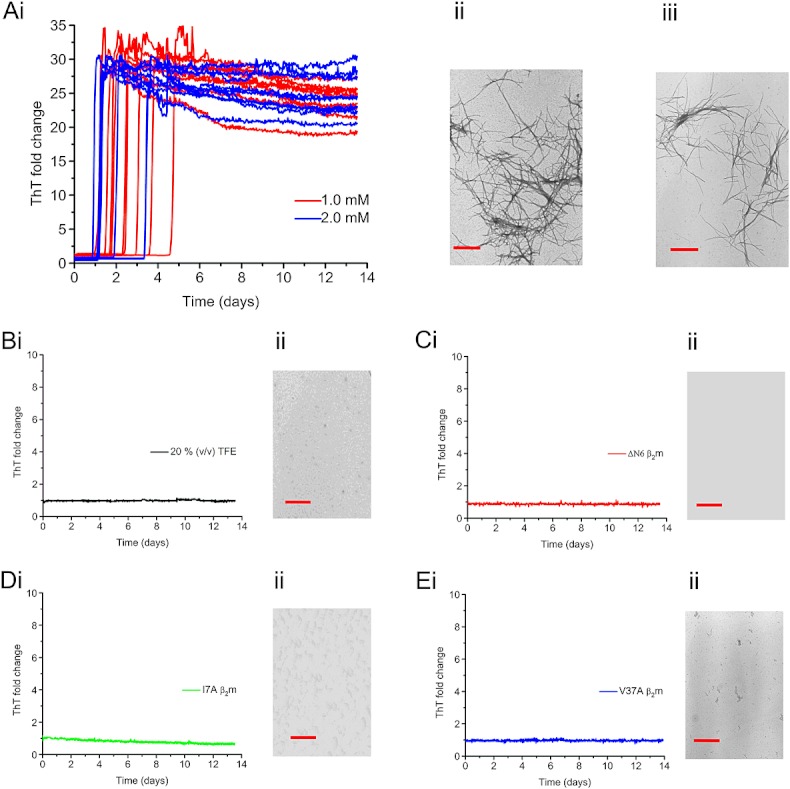
Fibril growth assays at pH 7.0, 37 °C with shaking at 200 rpm monitored by thioflavin-T fluorescence. A (i) wild-type β_2_m in the presence of 2 mM SDS (blue) or 1 mM SDS (red). Negative stain EM images of the fibrils resulting from incubation with (ii) 2 mM SDS and (iii) 1 mM SDS for 14 days are shown. B (i) wild-type human β_2_m in the presence of 20 % (*v/v*) TFE; (ii) the reaction end-point (14 days) observed by EM is shown. C (i) ΔN6 β_2_m; (ii) the reaction end-point (14 days) observed by EM is shown. D (i) I7A β_2_m; (ii) the reaction end-point (14 days) observed by EM is shown. E (i) I7A β_2_m; (ii) the reaction end-point (14 days) observed by EM is shown. Scale bars are shown in red and indicate 500 nm.

The data in Fig. 5 indicate that, by changing the solution conditions or sequence of β_2_m, the rate of EX1 HDX of the protein can be titrated over ~2 orders of magnitude. For example, a 70-fold decrease in the EX1 rate of exchange is observed when wild-type β_2_m is incubated in the presence of 500 mM trehalose compared with the same protein incubated in the presence of 20% (v/v) TFE. The wide range of HDX rates observed in this analysis provides an opportunity to determine the role of conformational dynamics in determining the amyloid potential of β_2_m monomers. To determine whether increasing the conformational flexibility of β_2_m results in an enhanced ability to nucleate fibril formation at neutral pH, conditions in which the rate of EX1 HDX kinetics is increased >5-fold were selected for further analysis (indicated by the dashed line at 0.05 min^–1^ in Fig. 5). For these samples, the ability of the protein to form amyloid fibrils was monitored under identical solution conditions (37 °C, 10 mM ammonium acetate/10 mM ammonium formate buffer, pH 7.0, ± additives, with mild agitation) by use of thioflavin-T fluorescence (see Experimental section). The fibril growth assay was performed for 14 days, at which point the presence or absence of fibrils was confirmed by negative stain transmission electron microscopy (EM). Of the 21 different solution conditions/variants studied using HDX, three conditions (wild-type β_2_m in the presence of 20% (v/v) TFE, 1 mM or 2 mM SDS) and four variants (ΔN6, P32G, I7A, V37A) resulted in an EX1 HDX rate that is increased >5-fold compared with that of the wild-type protein (Fig. 5). Of these seven conditions/variants investigated, however, only two conditions (wild-type β_2_m in the presence of 1 mM or 2 mM SDS) resulted in *de novo* fibril growth under the conditions and time-scales employed (Fig. 6). Interestingly, wild-type β_2_m in 2 mM SDS resulted in a very rapid HDX rate (23-fold compared with wild-type β_2_m in buffer alone) (Fig. 5) and these conditions resulted in rapid fibril formation (Figs. 6(A), (i) and (ii)). Addition of 1 mM SDS also resulted in *de novo* fibril formation in the 14-day time-scale employed with a similar lag-time to that observed with 2 mM SDS (Figs. 6(A), (i) and (iii)), but only produced a 10-fold increase in the rate of EX1 HDX (Fig. 5). The presence of 1 mM SDS resulted in a rate of EX1 HDX similar to that of V37A β_2_m (Fig. 5), yet only the former conditions led to fibril growth under the time-scale and conditions employed. None of the other conditions resulting in an enhanced rate (>5-fold) of EX1 HDX led to detectable fibril growth under the conditions employed (Figs. 6(B)–(E)). These analyses demonstrated that the probability of global unfolding measured by the rate of EX1 HDX kinetics did not correlate with the probability of amyloid fibril nucleation of β_2_m. Only for solutions containing 1–2 mM SDS did an increased rate of EX1 HDX correlate with *de novo* fibril formation. These conditions, therefore, resulted in unprotected species and solution conditions compatible with efficient fibril nucleation under the conditions explored.^44^

## DISCUSSION

It has been well documented that native β_2_m does not form amyloid fibrils in the absence of amyloid seeds *in vitro*, even when incubated at high concentration.^08^,^19^ These earlier data confirm that conformational rearrangement of the natively folded structure is necessary for fibril nucleation to occur,^16^,^33^ but whether fibrillogenesis requires rearrangement to a native-like species or more highly unfolded species, or both, under different solution conditions remained unclear.^9^,^33^,^47^ What is clear is that *in vitro*, at neutral pH (pH 7, 37 °C), the conformation(s) required to nucleate fibril formation of β_2_m are not populated sufficiently to allow assembly to proceed over an experimentally tractable time-scale. (Of course, this may not be the case *in vivo*, under conditions of different ionic strength and the presence of a range of other proteins and other biologically relevant factors, including glycosaminoglycans.^19^,^48^) Previously, it has been shown by use of HDX-MS that β_2_m bound to the MHC-1 heavy chain is conformationally stable and undergoes HDX by a pure EX2 mechanism *in vitro*, in marked contrast to the rapid dynamics that result in HDX occurring with an EX1 mechanism in monomeric β_2_m.^13^ Although it is not unusual for proteins to cycle through many different conformational states under native conditions depending on their Boltzmann weight,^49^ it is unusual for a thermodynamically stable protein to undergo exchange by an EX1 mechanism at pH 7 in the absence of denaturant, since the rapid refolding rate (k_f_) of most native proteins results in EX2 exchange kinetics at this pH.^50^ Here we have re-examined the EX1 HDX kinetics of human β_2_m by use of ESI-MS and, by comparison with the rate of global unfolding measured by denaturant jump experiments, revealed that the unprotected species identified by HDX-MS in buffer at pH 7.0 is the globally unfolded state. The data presented here also show that the frequency of visiting the unprotected (unfolded) state at pH 7.0 can be increased by incubation with additives such as SDS or TFE, or by alterations to the protein sequence. Interestingly, 5–20% (v/v) TFE and 0.5–10 mM SDS have been shown previously to enhance the rate of elongation of fibril seeds with β_2_m monomers,^40^–^44^ while the different variants studied result in varied abilities to nucleate fibril growth.^16^,^28^,^29^,^33^,^37^ Thus, ΔN6 is able to nucleate fibril formation efficiently at pH 7.0, I7A results in sparse fibril formation, and P32G, V37A and murine β_2_m are not able to nucleate fibril growth at this pH.^16^,^28^,^29^,^33^,^37^ Conditions that enhance the rate of EX1 HDX, therefore, do not enable the prediction of an ability to nucleate amyloid fibril formation. As well as reporting on the role of monomer dynamics in fibril growth, the results presented also highlight the importance of the solution conditions in determining fibril formation, with increased ionic strength and agitation often being required to drive fibril formation on a rapid (biochemically tractable) timescale even for a given pH and temperature.^51^ As the studies presented here were performed at low ionic strength, so that identical conditions could be employed for both the HDX and fibril growth assays, fibril nucleation was disfavoured. As a consequence, additives (e.g. TFE)^40^,^41^ and variants (ΔN6)^33^,^35^ known to enhance fibrillation at neutral pH failed to result in detectable fibril growth in 14 days in the volatile solvents employed here.

The highly dynamic nature of β_2_m, revealed here by HDX-MS, could influence fibril formation *via* two mechanisms. First, enhanced conformational dynamics might result in the exposure of buried hydrophobic residues and/or the exposure of regions of the polypeptide chain (main chain or side chains) with unsatisfied hydrogen binding potential that would enhance the probability of intermolecular associations required to nucleate fibril growth. Alternatively, increased conformational flexibility could lead to the enhanced population of partially unfolded species under native conditions. Although an increased frequency of unfolding to the globally unfolded state gives proteins a greater opportunity to visit aggregation-competent conformations, the solution conditions must also favour the continued population of these alternative conformations in order for bimolecular associations to occur and the species generated to be populated for time-scales commensurate with further fibril propagation. In the case of SDS and TFE it seems likely that although both additives cause frequent excursions to an unfolded state, SDS may endow thermodynamic stabilisation of an aggregation-competent conformation, whereas TFE does not offer this additional property. Thus, the action of SDS is 2-fold: to provide β_2_m with an increased opportunity to sample alternative conformations and also to stabilise the resulting aggregation-competent intermediate and/or oligomeric states *en route* to fibril formation. This suggests that the population of specific, aggregation-competent intermediates is a pre-requisite to the formation of oligomeric species and consequently higher order fibrillar structures. Although frequent transitions to the unfolded state of β_2_m are likely to allow increased kinetic sampling of aggregation-competent conformations, we have shown here that this is not sufficient to enable nucleation of β_2_m fibril formation at neutral pH. It has been suggested that conformational flexibility is dampened by evolution to reduce the potential risk of aggregation as described above.^52^ Here we show that monomeric β_2_m displays unusually high kinetic instability for an immunoglobulin domain, presumably because the evolutionary pressure on its sequence has been to enable tight binding to the MHC heavy chain.

## CONCLUSIONS

Using HDX-ESI-MS, this study has demonstrated that under conditions in which β_2_m appears to be compactly folded, the protein does visit a highly unprotected, globally unfolded conformational state. To explore the possibility that global unfolding determines the ability of β_2_m to aggregate and form fibrils at pH 7 *in vitro*, the HDX propensity of the protein was examined under different conditions. This involved analysing a range of protein variants known to have different thermodynamic stabilities and amyloid propensities, in addition to incubating wild-type β_2_m in the presence of a selection of additives, which had been reported previously to endow a range of effects on aggregation pathways. Together, these analyses demonstrate that the probability of global unfolding measured by the rate of EX1 HDX kinetics does not correlate with the probability of amyloid fibril nucleation of β_2_m.
